# How accessible are the websites of health services for people who have had a stroke?

**DOI:** 10.1186/s12939-025-02459-6

**Published:** 2025-04-24

**Authors:** Melita J. Giummarra, Eleanor Brown, Tanya A. Rose, Natasha A. Lannin, Brooke Parsons, Emma Power, On behalf of the Young Stroke Service team

**Affiliations:** 1 Department of Neuroscience, School of Translational Medicine, Monash University, 99 Commercial Road, Melbourne, VIC 3162 Australia; 2 University of Technology Sydney, Ultimo, NSW Australia; 3 Alfred Health, Melbourne, Australia; 4 Centre of Excellence for Aphasia Recovery and Rehabilitation, La Trobe University, Bundoora, VIC Australia; 5 Lived Experience Ambassador, Melbourne, Victoria Australia; 6 The Florey, Melbourne Brain Centre, Austin Hospital, Heidelberg, VIC Australia

**Keywords:** Stroke, Aphasia, Rehabilitation, Health services, Web accessibility, Accessibility, Internet access, W3C, WCAG

## Abstract

**Background:**

After sudden onset conditions (e.g., stroke), people commonly search for information online about healthcare and health services. Accessible websites are important for people with support needs, such as aphasia, to maximise their access to health service information. The accessibility of stroke-related health service websites against the Web Content Accessibility Guidelines (WCAG) and stroke-related access needs is not known. Therefore, the present study examined website accessibility of Australian health organisations, and their services, that provide post-stroke healthcare.

**Method:**

A cross-sectional descriptive study design was used to identify relevant health services in Victoria and South Australia. Organisation homepages and service webpages were assessed for WCAG errors and alerts using the WAVE® Web Accessibility Evaluation Tool. A 16-item stroke accessibility checklist was used to document accessibility issues for people with stroke-related communication, cognitive and sensory processing impairments. The checklist assessed webpage navigation, readability and formatting. Publication of an accessibility statement or policy on the website was recorded. Issues were classified according to perceivability, understandability, operability and robustness (POUR) domains.

**Results:**

A total of 185 webpages (126 homepages and 59 service-specific webpages) were evaluated against WCAG standards. Most webpages (*n* = 150, 81.1%) had at least one WCAG error (Median = 5 errors); the most prevalent being empty links that could not be read by a screen reader (*n* = 92, 49.7%). Checklist evaluations were completed for 105 webpages. Only 17 websites had an accessibility statement. Nearly all webpages had a reading level above Flesch-Kincaid Grade 6. Problems with readability, line height, font size, paragraph length, and bolding of key information were common. All had issues with ‘perceivability’ and ‘understandability’, and all but one website had operability issues. Only 10% of webpages contained robustness errors that could lead to compatibility issues across various browsers, devices, and assistive technologies. Government organisation websites had significantly fewer POUR accessibility issues than private sector sites.

**Conclusions:**

Health services should take concerted steps towards ensuring that their websites are accessible for all healthcare consumers, including people with language, cognitive and visual processing impairments, which are common after stroke. Online service information provides a key role in enabling stroke survivors to access information and make decisions about their healthcare.

**Supplementary Information:**

The online version contains supplementary material available at 10.1186/s12939-025-02459-6.

Stroke is a leading cause of disability in adults around the world [1], including for younger adults aged 35 to 54 years [2, 3]. Stroke can lead to impairments that often require ongoing access to healthcare. Difficulties include communication impairments (e.g., aphasia, a disorder characterised by impaired expression or comprehension of language), or physical impairments (e.g., vision impairments, hemiplegia, limb weakness), which can lead to restrictions in activities of daily living or social and economic participation [4]. Because aphasia fundamentally impairs language including reading and writing, stroke survivors with aphasia may have particular difficulty with accessing information from written text, images and videos on websites. However, stroke survivors may have support needs in multiple areas including communication, physical mobility, vision and cognition. Access to healthcare for all stroke survivors to improve functioning and psychosocial wellbeing remains key. Healthcare access is particularly critical for rehabilitation addressing longer-term goals such as return to work, financial stability, and maintenance of intimate and family relationships [5–7]. It is therefore important that people are able to find the right service at the right time to meet their needs [7–9].

Many people are now accustomed to searching for health-related information online [10], including people who have had a stroke [11]. People with language and communication difficulties post-stroke, in particular, frequently use digital technology and the internet to find information (e.g., about stroke, aphasia and support services), to support their rehabilitation, for leisure activities [12, 13], and to meet others (e.g., peer support) [14]. The availability and accessibility of digital resources has therefore become increasingly important for people with stroke who want to access information and make informed decisions about their healthcare [7, 15]. However, to date website accessibility problems have been a significant barrier to finding and understanding health information on the internet [16].

According to the social model of disability [17], the United Nations Convention on the Rights of Persons with Disabilities [18], and anti-discrimination legislation in many countries, all aspects of the community must be accessible. For instance, telecommunication systems and the internet must be designed to accommodate, circumvent or overcome participation difficulties experienced by people with disabilities [19]. The Web Content Accessibility Guidelines (WCAG) [20] is widely recognised as the international standard against which website accessibility compliance is assessed [21]. These guidelines comprise four main principles: (1) web content is easy for users to see, hear, and understand (*perceivable*); (2) users can navigate and operate website content using a variety of devices and input methods (*operable*); (3) web content is easy for users to understand and use, and predictable in its behaviour (*understandable*); and (4) web content is compatible with a wide range of browsers, devices, and assistive technologies (*robust*) [20].

Australian websites are expected to adhere to WCAG 2.0 or 2.1 Accessibility Standards (Level AA) [22]. While the WCAG has been internationally accepted, most health websites still do not meet the WCAG 2.0, Level AA standard. For instance, in an international study involving 25 countries, there were many errors in public health websites, particularly pertaining to “perceivable” attributes followed by “operable” and “understandable” characteristics [23]. Similarly, Australian studies have found frequent accessibility problems for 127 websites for mental health support services [24], and websites communicating information about COVID-19 [25]. It is apparent therefore, that although the WCAG guidelines are widely accepted they are not rigorously implemented on healthcare websites.

Several design features have been recommended to improve the accessibility of digital information for people with stroke in addition to the WCAG criteria. For instance, websites contain information written in active voice [26], with large text in a sans serif font [27–30], in short paragraphs [27, 28, 31], and at a Flesch-Kincaid Reading Grade of 6 or below [32]. Moreover, key words can be bolded, complex words should be explained [26, 30], and photographs or line drawings should be used to reflect key messages [28, 29, 31]. These recommendations, together with the WCAG criteria, are particularly focused on enhancing accessibility for people with sensory processing difficulties (> 50% of people)[7], cognitive impairments or fatigue (44% of people) [33, 34], and aphasia post-stroke (~ 33% of people) [33, 35].

However, to date, service-related information is often not provided in accessible formats, making it more difficult for people to independently find, access, understand and remember complex health service information on the internet after stroke. This barrier may negatively impact on their healthcare engagement and help-seeking behaviour [36].

While some studies have examined the accessibility of websites that provide information about stroke or aphasia, they have predominantly assessed readability [37, 38] and information quality [39–42], and none have examined adherence to both WCAG standards and stroke-related accessibility requirements. This is a major gap for this clinical population, making it difficult to know whether people with stroke-related impairments can decide whether a service is right for them. Therefore, we assessed whether health organizations and services offering stroke rehabilitation and healthcare meet international accessibility standards (WCAG) using the automatic WAVE evaluation tool, and whether they are compliant with stroke-specific accessibility needs through a customized accessibility checklist. Accessibility differences across service types were also explored to inform strategies to improve accessibility. In particular, we compared POUR accessibility elements across government, not-for profit and for-profit organisations to establish any differences in accessibility adherence across health sectors. We hypothesised that government services may have increased accessibility adherence due to greater expended alignment with government accessibility legislation and regulations serving marginalised citizens (e.g., Australian Government digital inclusion standard[43]).

## Methods

Ethics approval was obtained from the Monash University Human Research Ethics Committee (Project Number: 34654).

### Design

Cross-sectional descriptive study of health service websites identified as providing allied health and rehabilitation services to people who have had a stroke.

### Service eligibility

Services were included if they had a website and reported providing any rehabilitation or allied health hospital outpatient or community-based services to adults who have had a stroke. Services were identified from a larger project that was developing a directory of stroke services in Victoria and South Australia [44]. Eligible services included outpatient stroke or neurological rehabilitation clinics, community rehabilitation centres, community health centres, psychologists (including specialists, such as neuropsychology or health psychology), occupational therapists, physiotherapists, speech pathologists and social workers.

### Data sources

We assessed the organisation home page and, if present, the service webpages relevant to stroke (e.g., community health, stroke or neurological rehabilitation services) within the website. Homepages for all organisations were included as this is typically the first page visited that presents key information for the website and directs users to relevant pages. Homepages are therefore expected to be a good representation of whether the website incorporates design elements consistent with WCAG principles [45]. Where an organisation had service webpages in addition to their homepage, those additional stroke-related service pages were also included.

### Procedure

#### Identifying services and gathering service characteristics

Eligible services were identified using various methods, including searching previous publications; the National Health Service Directory (NHSD); professional association ‘find a provider’ listings or recommended services known to the Stroke Line team at The Stroke Foundation in Australia. As part of the larger Victorian and South Australian stroke service mapping study described in Giummarra et al.[see 45], a desktop audit and short key informant interviews and surveys were used to collect information about the services [44]. Characteristics reported in this study included: the classification of the service sector (e.g., public, private, not-for-profit), the service location, clinical specialties available at the service, and whether services were registered providers with the National Disability Insurance Scheme (NDIS). The NDIS is the national scheme in Australia that provides individualised funding for people with significant permanent disability to enable them to meet their life goals and aspirations, including therapy to build capacity[46]. The website evaluation included identification of whether information was provided on the website about stroke, communication difficulties or stroke-specific rehabilitation.

#### Website accessibility assessments

Websites were assessed during the period between January and August 2023. Altogether, the research team had expertise in stroke, aphasia, and digital communications, and the researchers completing the audits had backgrounds in clinical research and marketing and website analysis. Consistent with previous recommendations [47] multiple methods were used to assess the accessibility of the included websites: an automated accessibility evaluation tool, and a manually completed stroke accessibility checklist that was used to assess accessibility requirements for people with stroke who have impairments in language, cognition or visual processing. This approach was necessary because automated tools rarely evaluate all accessibility barriers experienced by people living with stroke [21, 48].

##### Automated testing

The WAVE evaluation tool was used to identify Level AA WCAG 2.1 accessibility problems, particularly for people with visual or mobility impairments. This tool was chosen as it has been used previously in website evaluations [49–51], and generates comprehensive reports against the WCAG guidelines. The WAVE tool identifies errors and alerts of potential errors, as well as the number of features, visual contrast errors and issues with structural elements within the webpage. It checks the use of HTML5 (a technology used for building the structure and organisation of websites), and the implementation of Accessible Rich Internet Application (ARIA). ARIA is a tool that can enhance website accessibility when used correctly, particularly for people who use assistive technologies like screen readers [52]. Alerts are potential errors that require human inspection to confirm whether they are true errors. The freely available WAVE extension for Chrome was used instead of the WAVE website to minimise the time required to complete each evaluation [53]. The total number of errors, alerts, Features, Structural Elements, and ARIA, as well as the specific errors (Table 3) and alerts (Table 4) from each report were recorded from the report from each webpage [52]. The WAVE evaluation was completed by the first two authors. Due to the automated nature of the WAVE application, the first and second authors each completed the analysis for allocated web pages until all pages were evaluated..

##### Manual checking

The stroke accessibility checklist was developed by the team for this study to document accessibility issues on websites that may affect people with language impairments due to aphasia and other cognitive or sensory processing impairments [33]. Sixteen criteria were included. However, two criteria (criteria 7 and 9) contained two unique elements. For example, reporting of numbers was assessed separately for (7a) small and (7b) large numbers as was whether headings and subheadings were bolded (9a) and distinct from content (9b). Therefore, the total number of potential issues for the 16 criteria was 18. The checklist was developed from issues previously identified as important for people with stroke accessing the internet [39]. An existing aphasia-specific tool, which had been developed for a previous scoping review [54] examining the accessibility of e-mental health programs, was extended to address other cognitive and sensory accessibility needs. In developing the checklist, we recognised that access difficulties may occur due to cognitive impairments (e.g., memory, attention, fatigue difficulties -navigating and following information), sensory difficulties (e.g., visual difficulties with small text) and physical difficulties (e.g., hemiplegia and difficulties using both hands to type). However, people with aphasia will have significant accessibility challenges as the medium of their impairments (reading, writing, auditory comprehension) are precisely the median of the website information provision (e.g., written text or online forms and videos). Therefore, issues around language access were the primary focus of the checklist. It is also notable that language-based supports also address needs of stroke survivors with other sensory processing difficulties such visual impairment (font size and spacing) and cognitive impairment (pop-ups and simpler reading level) and potential those with literacy difficulties or whose everyday language is not English. Additionally, evidence-based criteria were included based on recommendations for (1) webpage navigation; (2) written content and formatting; (3) graphic design and formatting of text, images, and videos; and (4) policies and plug-ins to support access (Supplementary Table 1) [28, 31, 55–57] that may support stroke survivors with various impairments (e.g., visual, cognitive, language, and motor difficulties).

*Navigation* issues included pop-ups, overlays, modals, and interstitials. These are website elements or pages that appear over the webpage, or when browsing between webpages, that typically require the viewer to interact with them in order to fully view or browse the webpage content (e.g., age verification or promotional material). For instance, since 2020 many health service websites present a notification pop-up box alerting users about COVID-19-related procedures at the health service. Moreover, an unstable interface, exemplified by slow loading times or page crashes, contributes to this reduction in navigation usability [31] (WCAG success criterion 3.2.1). *Written content* was assessed with respect to reading level, formatting and content. Readability was assessed using the Flesch Formula [58] (WCAG criterion 3.1.5), where lower Reading Ease scores indicate greater reading difficulty and Reading Ease scores below 30 are considered to require college-level education [59]. Materials for the general public should be presented at a Flesch-Kincaid Reading Grade level of 8 or below [60, 61]. However, for people with language (aphasia), cognitive and literacy difficulties, further modification of the reading level is required (i.e., a Flesch-Kincaid Reading Grade level 6 or lower and a Reading Ease score of ≥ 80) [60, 61]. Both the Flesch-Kincaid Reading Grade Level and Flesch-Kincaid Reading Ease scores measure text readability using similar data (e.g., word and syllable counts) and reporting both scores is common practice in accessibility research[37]. The Flesch-Kincaid Reading Grade reading level is referenced in international accessibility standards (WCAG) and government agencies in Australia[43], and United States (Federal Plain Language Guidelines[62]) rather than Reading Ease. Therefore, we include both measures in our study. Content was also assessed for paragraph length [27, 28, 31] (WCAG criterion 1.4.8); presentation of small numbers as figures [30] (WCAG criterion n/a); the use of common words only, or explanation of complex words [26, 30] (WCAG criterion 3.1.5); and the use of active voice [26, 63] (WCAG criterion n/a). The inclusion of *information* about aphasia or stroke anywhere on the website was assessed given that access to relevant information about a health condition and/or service will influence the ability to find and choose relevant health services after having a stroke [64–66].

Accessible *graphic design, text formatting and layout* as well as the suitability of images, and accessibility of videos were assessed. Text should be presented in a sans serif font [27–29] (WCAG criterion n/a), at line-height of at least 4 mm (24 px) [30] (WCAG criterion 1.4.12) and at a minimum text size of 14 (19px) or there should be a plug-in to adjust text size [28, 31, 67] (WCAG criterion n/a). Key words were considered more accessible if important information or terms were bolded [28, 54] (WCAG criterion n/a). It should be possible to zoom up to 200% without losing content or functionality [68] (WCAG criterion 1.4.4), and distinct headings should be linked to the content [31, 54] (WCAG criterion 1.3.1). In addition, images should be used to depict the core meaning of the content [28, 29, 54] (WCAG criteria n/a), preferably as line drawings or photographs [28, 29, 31], and videos should have synchronised captions that do not obscure the content [22] (WCAG criterion 1.2.2). Finally, the publication of an *accessibility policy* or statement anywhere on the website was recorded [66, 69, 70], and the availability of *accessibility control plug-ins* was also assessed. As manual evaluation against the checklist was significantly more time intensive than automated evaluation with the WAVE tool, we pragmatically focused on completing the stroke checklist evaluation of organisations and webpages that reported servicing young stroke survivors for our larger mapping study, and focused on the stroke or neurological rehabilitation or therapy-specific webpages where available. This focus resulted in a smaller number of webpages for analysis against the checklist than the WCAG WAVE tool evaluation, which was completed for all home webpages and service-specific webpages.

#### Rigour and reliability

As above the automatic WAVE evaluation was completed by the first two authors. The second and third authors completed the stroke accessibility checklist for a random sample ten webpages.

### Analysis

All data were collated in Excel (Office16 for Windows, Microsoft Corporation, Washington USA), and imported into Stata (Version 15, Texas, StatCorp, 2017) for analysis. Descriptive statistics were generated to characterise the prevalence and nature of accessibility problems, including comparisons across service sectors (i.e., government-funded services, private not-for- profit services and private for-profit services). The WCAG errors and stroke accessibility checklist criteria were classified according to the POUR criteria, with some criteria being allocated to more than one criterion as per previous research [23]. Accessibility according to each of the POUR criteria were compared across service sectors to help identify priorities for improving website accessibility. Exploratory analyses examined differences in accessibility issues between services using Chi-square tests. Consistency between assessors one and two were examined with Cohen’s Kappa and absolute observed agreement.

## Results

In this study, we evaluated whether stroke rehabilitation and healthcare websites met international accessibility standards (WCAG) using the WAVE tool and a customised checklist for stroke-specific accessibility needs. We then compared the POUR accessibility elements across government, not-for profit and for-profit organisations to establish any differences in accessibility adherence across health sectors.

### Included services

A total of 124 organizations were included (Table 1), with most based in major cities or inner regional areas (*n* = 113, 91.1%). These organizations spanned private for-profit (64), government (48), and not-for-profit/charitable sectors (12), with 67.7% offering two or more clinical specialties. The most common disciplines were physiotherapy (78.2%), occupational therapy (58.1%), psychology/neuropsychology (52.4%), speech pathology (53.2%), and social work (36.3%). While 79 organizations allowed payment via NDIS funding, only 53 were registered NDIS providers, and 18 registered providers did not require NDIS funding for stroke therapy, particularly in government health services where other public funding options were available.

**Table 1:** Characteristics of the services in the included organisations, *N* = 124

	Government organisations *n* = 48	Private not-for-profit or charitable organisation *n* = 12	Private for- profit organisations *n* = 64
Geographic location of organisations, *n(%)*			
Major Cities	16 (33.3)	11 (91.7)	48 (75.0)
Inner regional areas	25 (52.1)	1 (8.3)	12 (18.8)
Outer regional or remote areas	7 (14.6)	0	4 (6.3)
Clinical disciplines in the organisation^a^, *n(%)*			
Physiotherapy	47 (97.9)	10 (76.9)	40 (62.5)
Occupational Therapy	44 (91.7)	9 (75.0)	19 (29.7)
Psychology	28 (58.3)	7 (58.3)	21 (32.8)
Neuropsychology	14 (29.2)	1 (8.1)	13 (20.3)
Speech Pathology	37 (77.1)	10 (41.7)	19 (29.7)
Social work	36 (75.0)	5 (83.3)	4 (6.3)
NDIS registered provider, *n(%)*	35 (72.9)	5 (41.7)	31 (48.4)
NDIS budgets can be used to pay for services, *n(%)*	21 (43.8)	7 (58.3)	51 (79.7)

^a^The clinical disciplines were specifically recorded for services or clinics in organisations that were available to people who have had a stroke, and not for the organisation as a whole

### Included websites

A total of 185 webpages were evaluated against WCAG standards, including 126 homepages and 59 service-specific pages (Table 2). Some organisations had unique websites for different services (i.e. different specific services located within a single area health service, or different private hospitals administered by the same organisation), resulting in assessment of multiple homepages for those services. Checklist evaluations covered 105 webpages, including the homepage for 47 organisations, and a service page for 58 organisations. Only the homepage was assessed for some services, which were predominantly private organisations that described their service on the homepage.

**Table 2:** Characteristics of websites assessed, *N* = 185

	Government organisations *n* = 48	Private not-for-profit or charitable organisation *n* = 12	Private for- profit organisations *n* = 64
Total number of websites inspected, n^a^	50	12	66
Total number of webpages inspected	72	21	92
Total number of webpages inspected per organisation, N (%)			
One	24 (50.0)	3 (25.0)	40 (62.5)
Two	24 (50.0)	9 (75.0)	22 (34.4)
Four	0	0	2 (3.1)
Types of web pages inspected, n (%)			
Home pages	48 (66.7)	12 (57.1)	66 (71.7)
Service pages	24 (33.3)	9 (42.9)	26 (28.3)
Types of service page inspected, n (%)			
General services overview	0	0	6 (23.1)
Allied health services	3 (12.5)	1 (11.1)	1 (3.8)
Speech pathology	4 (16.7)	2 (22.2)	2 (7.7)
Neurological physiotherapy	0	0	5 (19.2)
Neuropsychology	0	0	1 (3.8)
Community services or rehabilitation services	11 (45.8)	3 (33.3)	4 (15.4)
Neurological therapy services	3 (12.5)	0	4 (15.4)
Stroke or brain injury services or information	3 (12.5)	3 (33.3)	3 (11.5)
Stroke characteristics anywhere on the website, n (%)			
Information about stroke or stroke rehabilitation	20 (40.0)	7 (58.3)	26 (39.4)
Information about aphasia, dysphagia or other communication difficulties is provided	12 (24.0)	8 (66.7)	11 (16.7)
There is an accessibility controls plug-in	14 (28.0)	2 (16.7)	2 (3.0)

^a^The number of websites is larger than the number of organisations as some organisations had multiple services, some of which had a separate website

Service-specific pages varied in content, including general service overviews (6), summaries of allied health services (5), specific disciplines (e.g., speech pathology, *n* = 8), and other service pages described community or rehabilitation services (18 websites), neurological therapy services (7 websites), or described therapy or information related to stroke or brain injury (9 websites). Information on stroke or stroke services appeared on 63.1% of websites, while communication difficulties were addressed on 24.2% of webpages.

### International accessibility standards (WCAG 2.1) using the wave assessment tool

One hundred and fifty webpages (81.1%) had one or more error with a median of five errors (Q1 = 1.0, Q3 = 13.0, max = 67) per webpage, Table 3. The most prevalent WCAG errors were empty links (i.e., a link containing no text that cannot then be read by a screen reader; *n* = 92, 49.7%) or buttons (i.e., interactive elements on a webpage that open a menu or execute a command; *n* = 40, 21.6%), missing alternative text for linked (*n* = 77, 41.6%) or embedded images (*n* = 41, 22.2%), and missing labels for form controls (*n* = 66, 35.7%).

**Table 3:** WAVE Errors on all webpages inspected that had ≥ 1 error, *N* = 150

			Number of errors	
Errors	Accessibility category	Number (%) of webpages with ≥ 1 error	Median (Q1, Q2)	Maximum number per webpage
Types of errors		150 (81.1)	2.00 (1.00, 3.00)	6
Broken ARIA menu	Robust	6 (3.2)	1.50 (1.00, 3.00)	3
Broken ARIA reference	Perceivable Robust	9 (4.9)	1.00 (1.00, 2.00)	3
Broken skip link	Robust	4 (2.2)	1.00 (1.00, 1.50)	2
Empty button	Perceivable Operable	40 (21.6)	2.00 (1.00, 5.00)	21
Empty form label	Perceivable Operable	11 (5.9)	1.00 (1.00, 3.00)	14
Empty heading	Perceivable	21 (11.4)	1.00 (1.00, 3.00)	16
Empty link	Operable	92 (49.7)	3.00 (1.00, 6.50)	49
Image button missing alternative text	Perceivable	1 (0.5)	1.00 (1.00, 1.00)	1
Image map missing alternative text	Perceivable Operable	2 (1.1)	1.50 (1.00, 2.00)	2
Language missing or invalid	Understandable	8 (4.3)	1.00 (1.00, 1.00)	1
Linked image missing alternative text	Perceivable Operable	77 (41.6)	2.00 (1.00, 3.00)	14
Missing alternative text	Perceivable	41 (22.2)	2.00 (1.00, 7.00)	43
Missing form label	Perceivable	66 (35.7)	1.00 (1.00, 3.00)	21
Missing or uninformative page title	Perceivable	2 (1.1)	1.00 (1.00, 1.00)	1
Multiple form labels	Perceivable	9 (4.9)	2.00 (2.00, 3.00)	8

A description of all errors identified by the WAVE tool is available online [52]

The following error types were not found on any webpages: Blinking content, Empty table header, Image map area missing alternative text, Invalid long description, Marquee, Page refreshes or redirects and spacer image missing alternative text

Nearly all webpages had one or more alert (*n* = 183, 98.9%) with a median of 16 alerts (Q1 = 6.00, Q3 = 27.00, max = 314) per webpage (Table 4). The most common alerts were redundant links (74.1% of webpages), no script element enabled (i.e., indicating that java script content would not be accessible; *n* = 119, 64.3%); very small text (*n* = 54, 29.2%) and suspicious link text (*n* = 48, 25.9%). There were issues with skipped heading levels for more than half of webpages (*n* = 100, 54.1%), including missing first level headings (*n* = 37, 20.0%), redundant title text (*n* = 58, 31.4%) or a possible heading (*n* = 53, 28.6%).

**Table 4:** WAVE Alerts on all webpages that had ≥ 1 alert, *N* = 183

			Number of alerts	
Alerts	Accessibility category	Number (%) of webpages with alerts	Median (Q1, Q2)	Maximum per webpage
Types of alerts, total		183 (98.9)	5.00 (3.00, 6.00)	10
A nearby image has the same alternative text	Perceivable	23 (12.4)	3.00 (2.00, 3.00)	5
Access key	Operable	4 (2.2)	1.50 (1.00, 2.50)	3
Broken same-page link	Robust	12 (6.5)	1.50 (1.00, 3.00)	13
Device dependent event handler	Operable	12 (6.5)	1.00 (1.00, 1.50)	4
Field set missing legend	Operable	2 (1.1)	3.00 (3.00, 3.00)	3
HTML5 video or audio	Perceivable	2 (1.1)	1.00 (1.00, 1.00)	1
Image with title	Perceivable	6 (3.2)	2.50 (1.00, 4.00)	13
Javascript jump menu	Operable	8 (4.3)	1.00 (1.00, 1.00)	1
Justified text	Perceivable	26 (14.1)	1.00 (1.00, 1.00)	32
Layout table	Perceivable	17 (9.2)	1.00 (1.00, 1.00)	4
Link to PDF document	Perceivable	6 (3.2)	1.00 (1.00, 2.00)	4
Long alternative text	Perceivable	10 (5.4)	1.50 (1.00, 4.00)	8
Long description	Perceivable	2 (1.1)	1.00 (1.00, 1.00)	1
Missing field set	Operable	4 (2.2)	1.00 (1.00, 1.00)	1
Missing first level heading	Perceivable	37 (20.0)	1.00 (1.00, 1.00)	1
No heading structure	Perceivable	1 (0.5)	1.00 (1.00, 1.00)	1
No page regions	Perceivable Operable	8 (4.3)	1.00 (1.00, 1.00)	1
No script element	Perceivable Operable	119 (64.3)	2.00 (1.00, 4.00)	45
Orphaned form label	Perceivable Operable	9 (4.9)	2.00 (1.00, 2.00)	8
Possible heading	Perceivable	53 (28.6)	3.00 (1.00, 8.00)	41
Possible list	Perceivable	1 (0.5)	1.00 (1.00, 1.00)	1
Redundant alternative text	Perceivable	30 (16.2)	3.00 (1.00, 6.00)	31
Redundant link	Perceivable Operable	137 (74.1)	4.00 (2.00, 10.00)	67
Redundant title text	Perceivable	58 (31.4)	4.50 (2.00, 10.00)	193
Select missing label	Perceivable	8 (4.3)	1.00 (1.00, 6.00)	6
Skipped heading level	Perceivable	100 (54.1)	1.00 (1.00, 2.00)	10
Suspicious alternative text	Perceivable	33 (17.8)	3.00 (1.00, 4.00)	40
Suspicious link text	Perceivable Understandable	48 (25.9)	1.00 (1.00, 4.00)	11
Tabindex	Operable	9 (4.9)	1.00 (1.00, 2.00)	4
Underlined text	Perceivable	27 (14.6)	2.00 (1.00, 6.00)	22
Unlabelled form control with title	Perceivable Operable	19 (10.3)	1.00 (1.00, 2.00)	4
Very small text	Perceivable	54 (29.2)	3.00 (1.00, 4.00)	107
YouTube video	Perceivable	3 (1.6)	1.00 (1.00, 1.00)	1

A description of all alerts identified by the WAVE tool is available online [52]

The following alert types were not found on any webpages: Audio/Video content, Flash content, Java applet present, unidentified Plugin present, and possible table caption (i.e., text appears to be a table caption, but is not a caption element)

### Stroke-related accessibility requirements using the stroke accessibility checklist

Webpages had an average of six (SD = 1.34) accessibility issues on the stroke accessibility checklist, out of a total possible score of 18, with no differences in the number of issues between for-profit, not-for-profit and government organisations; χ^2^(df = 2) = 0.99, *p* = 0.37 (Table 5). While nearly all webpages used a sans serif font, problems with readability, line height, font size, paragraph length, and bolding of key information were common. All webpages for government and private not-for-profit organisations had a reading level above grade 6, and only two private for-profit webpages had a reading level below grade 6, one of which only met this criterion as it had very little text. Overall, there were no differences in webpage readability (*p* = 0.89) or reading grade (*p* = 0.99) between sectors. Fifty-five (52.4%) webpages did not meet the minimum font size and did not have a plug-in to enable text adjustment, and 20% (*n* = 21) did not meet the minimum line height criteria for the main text on the webpage. With the exception of 13 webpages from private for-profit organisations, all other webpages could be zoomed to 200% without losing functionality. Most webpages were free of pop-up content. The majority of webpages (*n* = 92, 87.6%) had one or more paragraphs containing ≥ 80 words, and very few used bolding for key information (*n* = 10, 14.9%). The text on most webpages (*n* = 74; 70.5%) did not include numbers, so presenting numbers as both figures and words was rarely an issue. Fifty-four webpages (51.4%) included images, nearly all of which depicted the core meaning of the content. Eight webpages (7.6%) included videos, and the synchronised captions only blocked key information on one webpage. In terms of accessibility statements or policies published on websites, only 17 of105 (16.2%) webpages included this element.

**Table 5:** Prevalence of stroke and stroke-specific accessibility problems, *N* = 105 webpages

	WCAG category	Accessibility Category	Government organisations (*n* = 25 webpages)	Private not-for-profit or charitable organisation (*n* = 12 webpages)	Private for-profit organisations (*n* = 68 webpages)
Total number of issues per website, *Mean(SD) [range]*			5.72 (1.24) [1, 6]	6.00 (1.48) [2, 8]	6.16 (1.36) [2, 8]
3–4 issues			3 (12.0)	1 (8.3)	6 (8.8)
5–6 issues			17 (68.0)	9 (75.0)	35 (51.5)
7–10 issues			5 (20.0)	2 (16.7)	27 (39.7)
Stroke accessibility checklist, *N (%)*					
Anywhere on the website					
1. There is an accessibility statement or policy published or cited on the website	n/a	n/a	14 (28.0)	3 (25.0)	0
2. There is an accessibility controls plug-in	n/a	Operable	14 (28.0)	2 (16.7)	2 (3.0)
Service-specific webpages					
3. The webpage is not clear of pop-up content and has a stable interface	3.2.1 On Focus (Level A)	Operable	0 (0)	2 (16.7)	9 (13.2)
4. The Flesch-Kincaid Reading Grade Level is not at 6 or below	3.1.5 Reading Level (Level AAA)	Understandable	25 (100.0)	12 (100.0)	66 (97.1)
Readability level, *Mean(SD) [range]*			35.49 (8.11) [19.2, 49.9]	36.74 (14.60) [1.3, 54.4]	37.25 (17.26) [0, 90.9]
Reading Grade level, *Mean(SD) [range]*			12.05 (1.46) [9.8, 15.3]	12.06 (2.48) [9.3, 17.8]	11.99 (3.01) [5.7, 19.0]^a^
5. There are ≥ 80 characters per paragraph	1.4.8 Visual Presentation (Level AAA)	Understandable	16 (64.0)	12 (100.0)	64 94.1)
6. > 10% sentences are passive	n/a	Understandable	11 (44.0)	2 (16.7)	9 19.0)
7. (a) Small numbers are not presented as figures^b^	n/a	Perceivable Understandable	0	1 (50.0)	2 (11.1)
(b) Large numbers ('000 s) are not presented as both figures and words^c^	n/a	Perceivable Understandable	0	0	1 (100.0)
8. Bolding is not used to highlight important information, *n (%)*	n/a	Perceivable Understandable	22 (88.0)	10 (83.3)	62 91.2)
9. (a) Headings are not bolded^d^	n/a	Perceivable Understandable	13 (52.0)	5 (41.7)	34 (52.3)
(b) Subheadings are not linked to content^d^	1.3.1 Info and Relationships	Perceivable Understandable	2 (8.0)	2 (18.2)	8 (12.5)
10. A minimum line height is < 1.5 cm (24px) for the main text on the webpage	1.4.12 Text Spacing (WCAG 2.1, Level AA)	Perceivable Understandable	6 (24.0)	4 (33.3)	9 16.2)
11. There is no option for text adjustment, or the text is < 14 (19 px)	n/a	Perceivable Operable	8 (32.0)	5 (41.7)	42 61.8)
12. A sans serif font is not used^e^	n/a	Perceivable Understandable	1 (4.0)	0	1 (3.3)
13. The page cannot be zoomed to 200% without loss of content or function	Success Criterion 1.4.4 Resize text	Perceivable Operable Understandable	0	0	9 19.1)
14. Images are not line drawings or photos	n/a	Understandable	1 (12.5)	0	0
15. Images do not depict the core meaning of the content	1.3.1 Info and Relationships	Understandable	1 (12.5)	0	4 (9.8)
16. Videos do not include synchronized captions	1.2.2 Captions (Pre-recorded) (Level A)	Perceivable	2 (100.0)	1 (100.0)	4 (80.0)

*Note*: the methods for assessing each criterion are available online [52]

^a^One website of a private for-profit organisation had a Reading Grade level of 1.3; however, the page only included keywords about the service, opening hours and contact details, so the reading grade statistics excluded this outlier

^b^Small numbers not included in 14 webpages of government organisations, 10 webpages of private not-for-profit/charitable organisations, and 50 webpages for private for-profit organisations

^c^Large numbers were not on any webpages for government and private not-for-profit organisations, or on 67 (98.5%) of the webpages for private for-profit organisations

^d^There were no headings on three webpages of the private for-profit organisations, and no subheadings were used on one webpage for a not-for-profit organisation or any for-profit organisations

^e^Sans serif fonts were only partially used on seven websites for private for-profit organisations

### Rigour of service-specific webpage accessibility using the stroke accessibility checklist

There was substantial agreement between assessors across all service-specific webpage items (observed agreement = 86.3%, average kappa = 0.79, *p* < 0.005).

### Accessibility assessment across health sectors using the POUR accessibility elements

Our POUR assessment established the degree to which web content was *perceivable* (easy to see, hear and understand), *operable* (users can navigate with a variety of devices and input methods), *understandable* (content is easy to understand and use and predictable in its behaviour) and *robust* (content is compatible with a range of devices and browsers) [20]*.* We compared POUR elements across Government, not-for profit and for-profit organisations to establish any differences in accessibility adherence across health sectors.

All webpages had one or more issue in the perceivable and understandable domains (range: 1 to 13 issues), and 99.5% had one or more operable domain issue (range 0 to 8 issues), and 10.3% had robust domain issues (range: 0 to 1 issue). Websites for organisations in the Government sector had significantly fewer issues with all four POUR domains than services in the for-profit sector but did not differ significantly from the not-for-profit sector (Table 6). Altogether, there were problems with alternative text for images (a perceivable criterion) in more than half of webpages, with 103 (55.7%) webpages having one or more error, and 61 (33.0%) having one or more alert related to alternative text.

**Table 6:** Comparison of the number of perceivable, operable, understandable and robust errors in assessed webpages across service sectors

					*p*-values for post-hoc comparisons	
POUR criterion	(a) Government *N* = 72	(b) Private not-for-profit *N* = 21	(c) Private for- profit *N* = 92	p	a vs b	a vs c
**Perceivable**, Median (IQR)	4.00 (2.00, 8.00)	7.00 (4.00, 9.00)	8.00 (4.00, 9.00)	0.005	0.12	0.001
**Operable**, Median (IQR)	3.00 (2.00, 4.00)	3.00 (3.00, 5.00)	4.00 (3.00, 5.00)	< 0.001	0.051	< 0.001
**Understandable**, Median (IQR)	1.00 (1.00, 9.00)	9.00 (1.00, 10.00)	10.00 (1.00, 11.00)	< 0.001	0.17	< 0.001
**Robust**, number with one problem vs no problems	10 (14%)	5 (24%)	4 (4%)	0.013	0.28	0.03

*Notes*: there were 21 Perceivable criteria, 9 operable criteria, 14 understandable criteria and 3 robust criteria

## Discussion

The present study sought to examine the accessibility of the websites of organisations who provide rehabilitation and therapy services to adults who have had a stroke. We found that all webpages had accessibility issues and errors, particularly relating to whether the content was perceivable or understandable for people with sensory, mobility, cognitive or language-related disabilities. While the vast majority also had issues that would affect the operability of the webpage, only ten percent had errors that would make the content incompatible with browsers, devices, and assistive technologies (i.e., the *robust* accessibility domain). Finally, while all service types had similar rates of issues with readability and formatting of content, the webpages for government organisations had fewer POUR accessibility issues than those in the private sector, consistent with websites for mental health services [24]. This suggest that government services are not only setting better standards for the sector, but that their policies and procedures for website design and layout could also be used to support private services to improve their website accessibility. Finally, only 17 of the 105 webpages assessed had an accessibility statement or policy published or cited on their website, suggesting that demonstrating public commitment to accessibility may not be prioritised by all organisations.

The findings indicate that there are notable disparities in website design and adherence to the recognised accessibility standards outlined in the WCAG, and recommendations for people with stroke-related impairments. Some websites had so many issues in the design and formatting of the website, or specific webpages, that they likely may have been inaccessible for people with speech, language or cognitive problems after stroke, with the majority failing at least 5 to 10 of the stroke accessibility checklist criteria. The most common accessibility problems were attributable to perception and readability of information (e.g., poor or missing alternative text for images), and understanding of information, particularly captions on videos and text layout and formatting (e.g., presence and formatting of headings, bolding keywords).

Discernible trends emerged in the types of accessibility challenges on websites of government, not-for-profit, and for-profit organisations. While organisations in each sector had a similar numbers of accessibility issues, further exploration unveiled nuanced distinctions in the specific criteria where challenges manifested. Government organisations had fewer POUR issues overall, but it was apparent that many government organisation websites had issues related to the absence of accessibility controls plug-ins, potentially reflecting variations in compliance with the WCAG standards. In contrast, for-profit organisations often had issues with font and communication of information, including readability and size adjustments, emphasising that issues in the commercial sector may relate more to design-centric issues. Regarding WCAG alerts, the prevalence of potential redundant links and missing script elements across sectors poses critical challenges for users relying on assistive technologies. This was particularly problematic for private for-profit organisations where the rates of issues in the “robust” criteria were the highest. Government organisations faced a high incidence of skipped heading levels, suggesting potential difficulties for users navigating hierarchical content. These findings highlight the need for sector-specific interventions to enhance overall web accessibility.

Providing accessible information has been shown to support motivation and autonomy over treatment decision-making after stroke [71]. Moreover, Tomkins , Siyambalapitiya and Worrall [72] found that people with aphasia reported that their satisfaction or dissatisfaction with healthcare services was influenced by their experience of how information was exchanged, the ease and manner in which the service communicated with them more than other care aspects such as relationship building, therapy structure, or relevance. Providing service information in a way that it is accessible for people with stroke-related impairments should be a minimum standard of practice for rehabilitation services. However, the literature has continuously found that people with stroke, particularly those with aphasia, experience greater challenges in obtaining and understanding information about stroke [73], and when information is provided it is often not in an accessible format or medium [74]. Not surprisingly, when service-related information is inaccessible or not provided, it has a negative impact on service engagement and long-term help-seeking behaviour [36]. To enable people to self-direct their healthcare choices, reducing reliance on face-to-face or hospital-based communication, the accessibility of rehabilitation information on service websites must be improved. Indeed, making information more accessible to people with stroke-related impairments is likely to make that information more accessible to everyone in the community not just those with disabilities [75].

## Recommendations

The present findings have several implications for organisations that provide rehabilitation and therapy services for people who have had a stroke. Given so few organisations published an accessibility policy on their site, the development and/or communication of this type of statement will be an important first step forward in the commitment to making the healthcare sector more accessible to everyone in the community. Anecdotally, even services that had more accessibility controls and plug-ins on their websites reported in the larger project developing a Victorian and South Australian directory of stroke services that their allied health practitioners did not provide input into those accessibility functions. This is a missed opportunity given the expertise of those practitioners in adapting communication materials for people with a range of disabilities, particularly speech and language pathologists. Accessibility policies could include checklists and procedures that the organisation uses to ensure that their digital, online, printed, and physical environments meet the functional and communication access needs of all potential patients and stakeholders entering their service.

Organisations are able to test the accessibility of their websites before they are published against the WCAG standards using various free and paid-for automatic software tools (e.g., AChecker, Cynthia Says, EvalAcess, Fona, WAVE, WAQM). While most websites passed the “robust” accessibility domain, there were nonetheless issues that affected how people could interact with and navigate the websites; for example, interactive elements such as buttons, forms, and links are often not labelled correctly limiting accessibility for individuals who use assistive technologies while browsing the internet. Moreover, pop-ups negatively affect website accessibility, especially for people with cognitive impairments, and their use should be minimised.

Website visitors are likely to vary in their preferences for how they engage with health service information. Therefore, it is helpful when websites provide information in multiple formats including text, images, and videos. However, it is important that the information provided in each of these ways is accessible. First, text should be formatted in a way that it is easy to see and read (i.e., using a sans serif font that is large enough), and key information is easy to see and understand. For instance, important terms can be emphasised using bolding, and complex concepts can include an explanation. Because people with stroke may have cognitive and language difficulties, or even a background of low literacy, the readability level needs to be lower than that usually cited for the general public of grade 8 [60, 61]. Aiming for a Flesch-Kincaid Reading Grade level of grade 6 or below or a Reading Ease score of ≥ 80) [60, 61] will maximise accessibility for people with language (aphasia), cognitive and literacy difficulties. Altogether, these features relating to the text are relatively easy to change and will ensure that website visitors with low literacy or visual, cognitive or language-related impairments as well as those who are simply fatigued or busy understand the information being presented. Further, while photographs or line drawings that reflect key messages are helpful for people with aphasia to understand digital and printed information [28, 29, 31], it is important that websites provide alternative text to allow people with visual impairments to perceive the content of images as per the WCAG standards, as well as people who choose to use screen readers for other reasons. Alternative text should not include terms like “in this picture” and instead succinctly describe the meaning displayed in the image and any pertinent visual features, avoiding redundant information [76]. Services that participated in this study will receive a summary of study findings and strategies to address the issues commonly seen across websites and to maximise their website accessibility.

### Strengths and limitations

Although previous studies have evaluated more focused elements of websites (e.g., readability level or content quality [37, 38], this is the first study to present a rigorous and multifaceted evaluation of large number of websites for health services that are used by people following a stroke for both WCAG and stroke-specific accessibility adherence. We developed a comprehensive checklist to examine accessibility for people with visual, cognitive, and language-related impairments post-stroke alongside the assessment of the WCAG standards, which predominantly reflect visual or mobility-related impairments. As the primary aim of the study was to describe the alignment of webpages with stroke accessibility checklist items, we conducted only a preliminary reliability analysis of the overall checklist rating. Future research should focus on a more comprehensive psychometric evaluation and refinement of the checklist. Despite these strengths, the limitations of the study should be acknowledged. First, we did not undertake user testing, which is recommended for more comprehensive examination of the useability of the website [47]. Moreover, we did not assess the accuracy, relevance or usefulness of the stroke-related information presented on the webpages. We did not assess the use of common words or whether complex words were explained, which is an important aspect of text accessibility in the WCAG guidelines (3.1.5 Reading Level, Level AAA) and for people with aphasia and language disorders [26, 30]. We felt that this assessment would be better undertaken through consultation with users with a range of education levels and backgrounds, who could identify whether there are terms that they do not understand. Finally, we only used a single automated tool in one browser type (Chrome). Future studies should employ multiple tools given that different tools may yield varied results [77].

## Conclusion

In conclusion, the present study provides the first comprehensive insight into the pervasive challenges facing adults who have had a stroke when seeking health service information on the internet. These challenges are particularly detrimental for people who have difficulty understanding written text, images or videos due to language, cognitive, or visual impairments following stroke. The findings highlight that all health services that provide therapy for people after stroke, especially those in the private sector, need to do a better job of ensuring that their websites meet the WCAG standards and the communication access needs of their clients. We urge all health services to make a commitment to accessibility, critically appraise the accessibility of their websites, and engage therapeutic experts and patients in the design of their websites. This will help health services to foster an environment where individuals, regardless of their abilities, can more effectively navigate and engage with healthcare information.

## Supplementary Information

Supplementary Material 1.

## Data Availability

The data that support the findings of this study are available from resources available in the public domain; website URL listings are available on request from the corresponding author, E.P.
