# Supplementary material for: How accessible are the websites of health services for people who have had a stroke?
Source: Int J Equity Health. 2025 Apr 24;24:112. doi: 10.1186/s12939-025-02459-6 (PMC12020066; doi:10.1186/s12939-025-02459-6)
Supplement: Supplementary file 1 — Supplementary Material 1. [file 12939_2025_2459_MOESM1_ESM.docx]

# Supplementary Materials

**Supplementary Table 1**. Stroke accessibility checklist adapted from existing checklists and recommendations to characterise information needs and website accessibility for people who have had a stroke and who may have language or cognitive impairments.

|  |  |  |  | **Functional domain** | | |
| --- | --- | --- | --- | --- | --- | --- |
| **Accessibility Domain and Assessor** | **WCAG** | **Question (Y/N/NA)** | **References** | **Understanding language** | **Cognition (e.g., memory, processing speed)** | |
| **Content** | | | | | | |
| Judge | NA | 1. Is there stroke information on the website? | [69]* |  | | |
| Judge | NA | 1. Is there aphasia-specific information on the website? | [72, 73]* | **NA** | | |
|  |  |  |  |  |  |  |
| **Accessibility(2 criteria)** | |  |  |  |  |  |
| Judge | NA | 1. Is there an accessibility statement/policy published or cited on the website? | [72]* |  |  |  |
| Judge | NA | 1. Is there an accessibility control plug-in? |  |  | | |
| **Navigation (1 criterion)** | | | | | | |
| Judge | 3.2.1 On Focus (Level A) | 1. Is the website clear of pop-up content and has a stable interface? | [33]* | X | | X |
| **Written content (4 criteria, 5 measures)** | | | | | | |
| Microsoft Word | 3.1.5 Reading Level  (Level AAA) | 1. Is the Flesch-Kincaid Reading Grade at 6 or below? | [34]* | X | |  |
| Microsoft word/Judge | 1.4.8 Visual Presentation  (Level AAA) | 1. Are there 80 or fewer characters for paragraph width? | [29, 30, 33]* |  | | X |
| Judge | NA | 1. Is an active voice are used throughout the content? | [28]* | X | |  |
| Judge | NA | 1. (a) Are smaller numbers presented as figures? (b) Are large numbers ('000s) presented as both figures and words? | [32]* | X | |  |
| **Design/formatting (6 criteria, 7 measures)** | | | |  |  |  |
| Judge | NA | 1. Is bolding used to highlight important information? | [30, 56] | X | | X |
| Judge | 1.3.1 Info and Relationships | 1. (a) Are headings bolded? (b) Are headings/ subheadings distinct and linked to content? | [33, 56] | X | | X |
| Inspect in Google Chrome, Computed values | 1.4.12 Text Spacing  (WCAG 2.1  Level AA) | 1. Is a minimum value of 1.5 (4mm, 24px) line-height for the main paragraph content? | [32]* | X | |  |
| Inspect in Google Chrome, Computed values | NA | 1. Is there an option for text adjustment? If not, is text in size 14 (19px) at the minimum? | [30, 33, 70]* | X | |  |
| Inspect | NA | 1. Is a sans serif font (e.g., Arial or Calibri) used? | [29-31]* |  | | X |
| Judge/Chrome zoom | Success Criterion 1.4.4 Resize text | 1. Can the page be zoomed (200%) without loss of content or functionality (e.g., text does not overlap)? | [71]* | X | |  |
| **Images (2 criteria)** | | | | | | |
| Judge | NA | 1. Are images either line drawings (e.g., icons) or photographs? | [30, 31, 33]* |  | | X |
| Judge | NA | 1. Do the images depict the core meaning of the content block? | [30, 31, 56] | X | | X |
| **Multimedia (1 criterion)** | | | |  | |  |
| Judge | 1.2.2 Captions (Pre-recorded)  (Level A) | 1. Do videos offer synchronised captions? | [24]* | X | |  |

* These criteria were not included in the checklist used by Clunne SJ, Ryan BJ, Hill AJ, Brandenburg C and Kneebone I [56]
